# Evaluation of Symptoms and Prevention of Cancer in Menopause: The Value of Vulvar Exam

**Published:** 2016-11-01

**Authors:** AR Palumbo, C Fasolino, G Santoro, V Gargano, M Rinaldi, B Arduino, M Belli, M Guida

**Affiliations:** 1 University of Medicine and Surgery, Unit Obstetrics and Gynecology, Salerno, Italy; 2 LILT (Lega Italiana per la Lotta contro i Tumori), Avellino, Italy; 3 Department of Obstetrics and Gynecology, A.U.O.” San Giovanni di Dio e Ruggi d’Aragona”, via S. Leonardo, Salerno, Italy

**Keywords:** vulvar cancer, cancer prevention, vulvar examination

## Abstract

Vulvar and vaginal atrophy (VVA), is a chronic medical condition experienced by postmenopausal women, with prevalence estimated ranging from 10% to 50% [1]. VVA is characterized by a constellation of symptoms, that may affect daily activities, sexuality, relationships, and quality of life [3]. Early recognition and effective treatment of VVA may enhance sexual health and the quality of life of women and their partners. Some vulvar conditions such as lichen sclerosus are more prevalent in the postmenopausal years. Lichen sclerosus has been suggested as a precursor of Vulvar squamous cell carcinoma. The vulvar exam in post-menopausal women plays an essential role in prevention of cancer because it allows to identify women who should undergo vulvar skin biopsy in order to early detect pre-neoplastic lesions for early diagnosis of cancer of the vulva.

## I. INTRODUCTION

Vulvar and vaginal atrophy (VVA), is a chronic medical condition experienced by post-menopausal women, with prevalence estimated ranging from 10% to 50% [1]. VVA, resulting from the loss of estrogen stimulation, is characterized by thinning of the epithelial lining of the vagina and lower genitourinary tract, by loss of vaginal elasticity, by vaginal dryness and by an increase of vaginal Ph [2], determining a significant impact on sexual health and quality of life. VVA is characterized by a constellation of symptoms, such as vaginal dryness, burning and irritation, lack of lubrication, dyspareunia, dysuria, and urinary urgency that may affect daily activities, sexuality, relationships, and quality of life [3]. Unlike hot flushes that usually resolve over time, VVA has a chronic progressive nature throughout the menopausal transition and beyond. The presence and severity of symptoms are variable, from mild discomfort to great impairment, depending also on age, time and type of menopause, parity and vaginal delivery, frequency of coital activity, cigarette smoking and certain medical conditions [4].

The REVIVE survey was an online evaluation of postmenopausal women in the US. The recently published REVIVE (REal Women’s VIews of Treatment Options for Menopausal Vaginal ChangEs) survey, offers many insights into the impact of VVA symptoms on women’s lives. The most commonly reported VVA symptoms among post-menopausal women were vaginal dryness (55%), dyspareunia (44%), and local irritation (37%) [5]. While the clinical picture of VVA and other conditions subsequent to menopause have been thoroughly described, there has been little quantitative research on the association of VVA with other disorders, such as the structural modifications of the vulvar skin.

Vulvar itching is the main symptom of Lichen sclerosus of the vulva, a chronic dermatologic condition characterized by pruritus, inflammation and tissue scarring, most often affecting post-menopausal women. [6] Additionally, women may report cracking or bleeding of the vulvar skin and perianal area.

Patients with lichen sclerosus especially those with squamous cell hyperplasia have an increased risk of vulvar malignancy. The increased risk of developing squamous cell carcinoma is approximately 5 percent in patients with lichen sclerosus [7]. Accurate diagnosis is essential for effective treatment. The risk of progression to malignancy associated with some of these diseases dictates long-term surveillance [8].

This paper discusses implication of VVA symptoms on women’s lives and stresses value of vulvar exam in early identification of pre-neoplastic lesions in postmenopausal women.

## II. METHODOLOGY

Our study population included 44 sexually active postmenopausal women 50 to 75 years old, recruited from LILT ambulatory in Avellino, Italy. We administered the Vulvovaginal Symptoms Questionnaire (VSQ), a previous validated instrument to post-menopausal women.

The first six questions of the questionnaire comprise the symptom subscale (itching, burning, dryness, dyspareunia, urinary frequency, urinary urgency). Women who answered “Yes” to any of the first six symptom questions were considered to have vulvovaginal symptoms.

Eligible participants were all Italian women in post-menopause, which is defined as the presence of amenorrhea for at least 12 months. These women consulted an outpatient gynecological service for a routine gynecological examination. Among eligible women, only those who signed an informed consent were enrolled into the study. All women were subjected to a medical interview through which demographic information, personal and family medical history and comorbidities were recorded. Subjective symptoms considered were: vaginal dryness, itching, burning, dysuria and dyspareunia.

Objective signs taken into consideration were: thinning of vaginal rugae, bleeding at slightest touch, bleeding at intense touch and presence of petechiae.

## III. RESULTS

Post-menopausal women included in Our study reported at least one vulvovaginal symptom. The most common symptom reported is dryness 88.63% (n/N= 39/44), whereas the symptoms recorded less frequently are urinary frequency 38.63% (n/N= 17/44) and urinary urgency 34.09% (n/N=15/44). Vulvovaginal symptoms have important repercussions on the sexual sphere, in fact the symptom of dyspareunia was recorded in 54.54% (n/N= 24/44) of sexually active post-menopausal women. Sizeable proportions of women with vulvovaginal symptoms report itching in 52.27% (n/N=23/44) and burning 56.81% (n/N=25/44). *(Graphic 1: Incidence of VVA symptoms)*

Objective examination of the external genitalia allowed to record several alterations of the vulvar skin. Alterations of the vulvovaginal mucosa were classified into five categories: normal mucosa, thinned mucosa, bleeding at the slightest touch, bleeding at intense touch and presence of petechiae. (*Graphic 2: Alterations of vulvovaginal mucosa. Comparing two groups: women with no itching and itching)*

In the subgroup of post-menopausal women presenting itching 52.27% (n/N=23/44), only 26,08% (n/N=6/23) of sexually active post-menopausal women have normal mucosa, whereas 73,91% (n/N= 17/23) show alterations of the vulvar skin. *(Graphic 3: Alterations of vulvovaginal mucosa in the subgroup of women presenting itching)*

The results of the evaluation of the external genitalia show the presence of thinned mucosa in 47,05% (n/N= 8/17), bleeding at slightest touch in 11,75% (n/N= 2/17), bleeding at intensive touch in 29,41% (n/N= 5/17) and presence of petechiae in 11,75% (n/N= 2/17). *(Graphic 4: Pathological aspects of vulvovaginal mucosa in the subgroup of women presenting itching)*

## IV. DISCUSSION

Pathogenesis of VVA symptoms is reflected in the reduction in the level of estrogenic hormones, typical phenomenon which occurs in the period of menopause. Estrogen receptors, alpha and beta, are expressed throughout the squamous epithelium, connective tissue and smooth muscle of the vulva, vagina, urethra, and bladder trigone and are critical for mediating numerous biochemical and physiologic functions during a woman’s reproductive years [9]. With loss of estrogen stimulation, profound changes occur within the vulvovaginal and urogenital mucosa. In the dermal layer, collagen fibers fuse and undergo hyalinization, whereas the elastin fibers fragment. The result of these changes is an overall loss of mucosal elasticity and the vaginal epithelium thinning [10].

Estrogen-induced parakeratosis of the vulvar squamous epithelium progressively decreases after menopause and is rarely seen in older women. The vagina loses its rugae, and there is a shortening and narrowing of the vagina. The mucosa of the vagina, introitus, and labia minora become thin and pale. Surrounding vascularity, highly estrogen dependent, also decreases. [11]

Vulvar disease in the post-menopausal age group is relatively common. Some vulvar conditions such as lichen sclerosus are more prevalent in the post-menopausal years. Often more than one condition is present at the same time [8]. Lichen sclerosus of the vulva is a chronic dermatologic condition characterized by pruritus, inflammation and tissue scarring [6]. The distribution of skin changes may involve the perianal skin, and scarring can cause fusion of the labia minora to the labia majora and flattening of the clitoral hood, which may result in immobility [12].

Physical examination is important for differentiating lichen sclerosus from other causes of vulvar pruritus, such as lichen planus, lichen simplex chronicus, atrophic vaginitis, irritant contact dermatitis, eczema, psoriasis, vulvovaginal candidiasis and skin cancers (e.g., vulvar intraepithelial neoplasia and extra-mammary Paget disease) [13].

Patients with lichen sclerosus, especially those with squamous cell hyperplasia, have an increased risk of vulvar malignancy and should be monitored accordingly. The increased risk of developing squamous cell carcinoma is approximately 5 percent in patients with lichen sclerosus. [7].

Recognition and treatment of these vulvar conditions is important for symptom relief, sexual function, prevention of progressive vulvar scarring, and to provide surveillance for associated vulvar cancer [14].

Vulvar cancer is a relatively rare gynecologic malignancy with an annual incidence in developed countries of approximately 2 per 100,000 women. Vulvar squamous cell carcinoma (VSCC) has two etiological pathways: a high risk human papillomavirus (HPV)-dependent route, which has usual vulvar intraepithelial neoplasia (uVIN) as a precursor lesion, and an HPV-independent route, which is associated with differentiated VIN (dVIN), lichen sclerosus, and genetic alterations, such as TP53 mutations [15].

Most VSCCs originate in intraepithelial lesions, named vulvar intraepithelial neoplasia (VIN), that precede the development of VSCC by a variable period of time. Epithelial disorders are found adjacent to VSCC in 50–70% of patients. Most of these intraepithelial changes appear to have some degree of dysplasia [16].

Lichen sclerosus (LS) has also been suggested as a precursor of HPV-independent VSCC, but the mechanism of carcinogenic progression from LS has not been fully delineated. The association between the two entities has been established mainly because the majority of vulvar cancers will have lichen sclerosus, squamous cell hyperplasia or differentiated VIN in the adjacent epidermis [17] and because some longitudinal cohort studies have shown that women with LS have a significantly higher risk of developing VSCC.[18]

It is clear the value of the vulvar exam to identify the presence of skin changes associated with the presence of a preneoplastic lesions.

Cancer Research UK publishes the symptoms of vulvar cancer [19]

- a lasting itch
- pain or soreness
- thickened, raised, red, white or dark patches on the skin of the vulva
- an open sore or growth visible on the skin
- burning pain when you pass urine
- vaginal discharge or bleeding
- a mole on the vulva that changes shape or colour
- a lump or swelling in the vulva

The presence of these alterations should be an alarm bell and must lead the gynecologist to perform a vulvar skin biopsy. Vulvar biopsy is the only method to get a histopathological diagnosis when there is a vulvar lesion of uncertain significance, or in the presence of persisting symptoms, such as vulvar irritation or itching [20].

## V. CONCLUSION

VVA symptoms negatively affect the quality of life of menopausal women, complicating interpersonal relationships and married life. Early recognition and effective treatment of VVA may enhance sexual health and the quality of life of women and their partners.

Lichen sclerosus has been suggested as a precursor of HPV-independent VSCC. The etiology of lichen sclerosus is multifactorial; therefore, patient education, behavior modification, and regular follow-up with an experienced clinician are essential to ensure effective control of patient symptoms and management of the skin condition. The vulvar exam in post-menopausal women plays an essential role in the early detection of pre-neoplastic lesions. In presence of injuries such as itch, pain, raised patches and bleeding, the gynecologist must start a therapeutic treatment. If during the follow-up of skin lesions there is a lack of response to treatment or a progression of lesions, the gynecologist must proceed with the execution of a biopsy of the vulvar skin. The recognition of vulvar lesions is important for the prevention and early diagnosis of cancer of the vulva.

## Figures and Tables

**Graphic 1:**
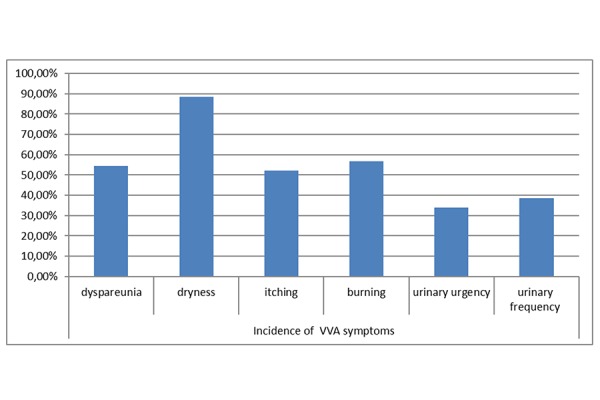
Incidence of VVA symptoms

**Graphic 2:**
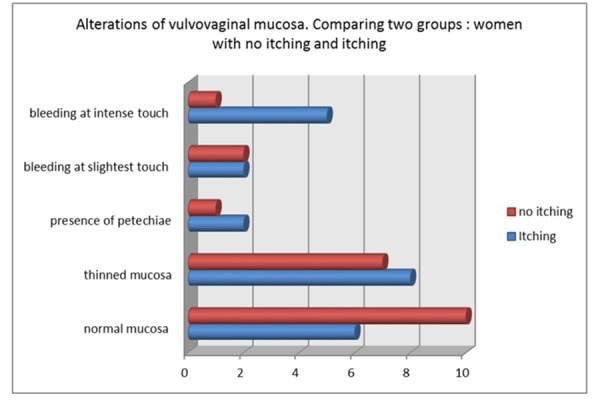
Alterations of vulvovaginal mucosa. Comparing two groups: women with no itching and itching

**Graphic 3:**
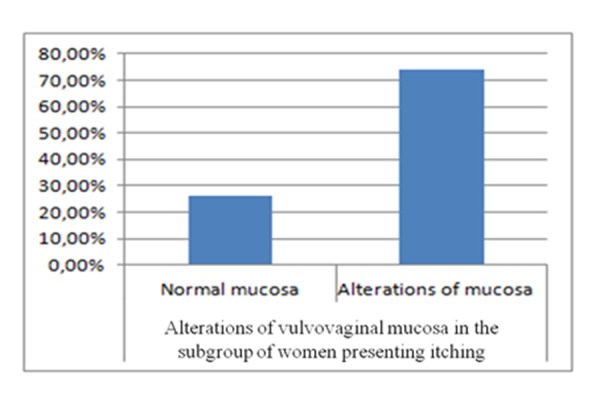
Alterations of vulvovaginal mucosa in the subgroup of women presenting itching

**Graphic 4:**
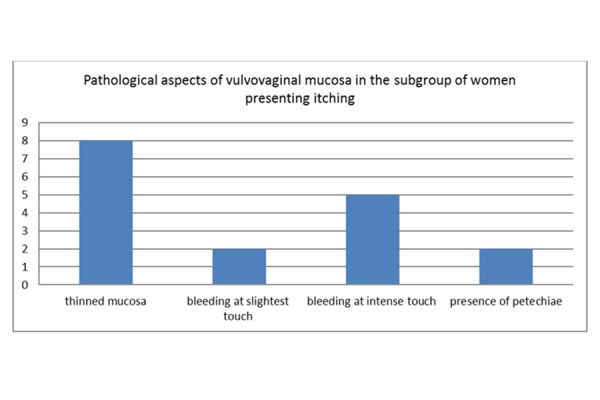
Pathological aspects of vulvovaginal mucosa in the subgroup of women presenting itching
